# Sulphuric Ether Used in the Reduction of Two Cases of Dislocation of the Humerus

**Published:** 1847-08

**Authors:** F. H. Hamilton

**Affiliations:** Buffalo


ART. IV.—*Sulphuric Ether used in the reduction of two cases of dislocation of the humerus.* Reported by Dr. F. H. Hamilton, Buffalo.

1*st Case.*—John Harrington, aged 50; of a herculean frame and with powerful muscles, had his left humerus dislocated into the axilla on the sixth of July. On the 16th, ten days after the accident, he called upon me, and in presence of Drs. John Trowbridge, Garvin, Dudley, Harvey and others, I made attempts at reduction as follows:

*First* attempt.—Applied the compound pulleys, and extended the arm with as much force as would be prudent, fifteen minutes—arm at right angles with the body—rotating the arm occasionally during this time.

*Second.*—Extended the patient upon the floor, and drew the arm in line of body, having my heel in the axilla (without pulleys).

*Third.*—Patient lying in the same position, I pulled the arm at right angles with the body.

*Fourth.*—Patient still extended, I adopted Malgaigne's method, pulling upward, with my foot upon the top of the shoulder.

*Fifth.*—Patient seated on a chair, I drew the arm down over my knee, which rested in the axilla.

*Sixth.*—Bled him twenty-four ounces, and until partial syncope was induced, and then tried again the *second* position.

*Seventh.*—Repeated the *first* attempt during ten minutes, and now, while the extension was kept up fully, I made him respire sulphuric ether until he was completely intoxicated and insensible.

*Eighth.* Before the influence of the ether had began to subside, the band to which the pulleys were attached broke, I then brought the axilla over the knee, (*fifth* position) and the bone was made to resume its place with great ease. The relaxation of his muscles was now very complete. John said the ether was the best grog he ever took.

Imediately after reducing this arm, I went with Dr. Dudley to his office to witness the reduction of the same luxation in another patient.

*Case* 2d.—Peter Starr, aged 45, Dutchman, very muscular, fell this morning on the upper end of humerus and produced a dislocation downwards into the axilla—right arm.

Dr. Dudley with two other men had soon after made attempts at reduction, but without success. Dr. Dudley then determined to use the pulleys, and, at my suggestion, to assist their operation by the ether.

Dr. D., applied the pulleys to the patient at eleven o'clock, four hours after the accident, and immediately Dr. Harvey commenced the administration of the ether; in about one minute after its full effects were obtained, the bone slipped into its place.

The patient said he was as if asleep, and that he knew nothing of it: yet, during the extension he drew his face, as though suffering pain.

